# **Community Health Workers Bring Cost Savings to Patient-Centered Medical Home**s

**DOI:** 10.1007/s10900-017-0403-y

**Published:** 2017-07-10

**Authors:** Maurice L. Moffett, Arthur Kaufman, Andrew Bazemore

**Affiliations:** 1 Department of Family and Community Medicine, University of New Mexico, Albuquerque, NM USA; 2 Office for Community Health, University of New Mexico, 2400 Tucker, NE, Albuquerque, NM 87131 USA; 3 The Robert Graham Center, Washington, DC USA

**Keywords:** Community health workers, Patient-centered medical homes, Cost savings

## Abstract

The Patient-Centered Medical Home (PCMH) model demonstrated that processes of care can be improved while unnecessary care, such as preventable emergency department utilization, can be reduced through better care coordination. A complementary model, the Integrated Primary Care and Community Support (I-PaCS) model, which integrates community health workers (CHWs) into primary care settings, functions beyond improved coordination of primary medical care to include management of the social determinants of health. However, the PCMH model puts downward pressure on the panel sizes of primary care providers, increasing the average fixed costs of care at the practice level. While the I-PaCS model layers an additional cost of the CHWs into the primary care cost structure, that additional costs is relatively small. The purpose of this study is to simulate the effects of the PCMH and I-PaCS models over a 3-year period to account for program initiation to maturity. The costs and cost offsets of the model were estimated at the clinic practice level. The studies which find the largest cost savings are for high-risk, paneled patients and therefore do not represent the effects of the PCMH model on moderate-utilizing patients or practice-level effects. We modeled a 12.6% decrease in the inpatient hospital, outpatient hospital and emergency department costs of high and moderate risk patients. The PCMH is expected to realize a 1.7% annual savings by year three while the I-PaCS program is expected to a 7.1% savings in the third year. The two models are complementary, the I-PaCS program enhancing the cost reduction capability of the PCMH.

## Introduction

The seven principles defining the Patient-Centered Medical Home (PCMH) barely addresses the social determinants of health (SDH) which play a far greater role in health and premature mortality than does the health care system [1]. The PCMH has been widely promoted and recognized as a model for achieving effectiveness, equity and efficiency of care. However, the model demonstrates only a modest cost savings. Would addressing SDH increase cost savings? Community health workers (CHWs) offer a path towards contextual and team-based care and a means of addressing the SDH. CHWs are culturally and linguistically competent individuals who usually live in the communities served and possess skills in addressing SDH. CHWs can be integrated into primary care practices either supporting the PCMH model or serving as a structural alternative to meet PCMH goals [2, 3].

Studies of PCMH showing cost savings have tended to link returns to reduced emergency department visits and avoided preventable hospitalizations at the patient level [4, 5]. However, the overall cost analysis must address the effect of the PCMH model on resource use at the practice level, where an important consideration, for example, is the establishment of effective panel sizes. Case studies suggest that successful PCMH implementation requires reduction in panel sizes per primary care provider, for instance from 2300 to 1300 [6]. The result is that while the cost measured at the patient level decreases, the overall cost of managing the practice population rises.

Most studies find the bulk of PCMH cost savings derived from care of the small number of high-risk, paneled patients. The studies show fewer effects of the PCMH model on the larger, moderate-utilizing patients and little evidence of effect on low-risk, paneled patients, who form the great majority of any practice population [6]. Studies on the cost effects of CHWs usually focus on the navigation of high-risk patients away from the emergency department and towards primary and specialty services [7, 8]. Little is written about the impact of the PCMH or CHWs models on practice-level costs.

The Integrated Primary Care and Community Support (I-PaCS) CHW model was developed to complement PCMH. Specific interventions include screening patients for the social determinants of health, connecting patients with resources to address those social determinants, assisting patients to navigate health and social services systems, and empowering patients to be active members in the process. Each patient is screened for adverse social determinants; those who screen positive are referred to the CHW for services and depending on their health and social needs, receive varying levels of CHW support. All the data collected from the screening tools is then compiled and used in summary form to better understand the broad social issues impacting the community served by the clinic, informing broader community health improvement strategies led by those working on policy efforts.

## Methods

We used published literature to estimate the impact of PCMH and comprehensive CHW models such as I-PaCS on cost containment. To create this preliminary estimate of cost savings for the two models, we made several assumptions. First, we assumed that the cost savings should be evaluated from a practice perspective. Second, we assumed that the programs serve a Medicaid-managed care population that exists in New Mexico that is composed of 5% in the high risk/high cost category, 15% at moderate risk/moderate cost, and the remaining 85% in good health at low risk/low cost. The base-case and service categories for inclusion in the model are from the CMS Office of the Actuary [9]. Estimates of the cost effects of the PCMH and I-PaCS CHW model was extracted from the Patient-Centered Primary Care Collaborative annual report and from direct CHW cost studies.

## Results

The actuarial estimates for the PCMH calculations in the Table, based on available literature, anticipate at 12.6% decrease in the inpatient hospital, outpatient and emergency services for high-risk/high cost patients, a 7% decrease in the inpatient hospital, outpatient and emergency services for patients receiving clinically integrated support and no expected decreases for the lowest-risk patients. For primary and specialty care, we modeled a 2.7% decrease in the costs for only the high-risk/high-cost patients. For outpatient services, we predicted that costs would decrease approximately 70% for high-risk patients, 40% for moderate-risk patients and 0% for the healthy population. Outpatient prescription drug costs have two competing effects. The first increases drug costs due to increased medication management and improved medication adherence and the second decreases drug costs due to reduced reliance on narcotics and improvement in medication therapy management in patient over-prescription from using multiple specialists for chronic conditions. We anticipate a net increase in use at 2% (Table 1).

**Table 1:** Expected impact and magnitude of cost effect by service category

Service category	PCMH (%)	Comprehensive CHW (%)
Inpatient hospital	−1.0	−19.0
Outpatient hospital	−1.0	−19.0
Emergency services	−3.4	−19.6
Professional primary care	+0.5	+20.0
Professional specialty care	+0.5	+10.0
Laboratory services	0.0	+10.0
Other (capture costs)	0.0	+5.0
Prescription drugs (outpatient)	+2.0	+2.0

**Fig. 1:**
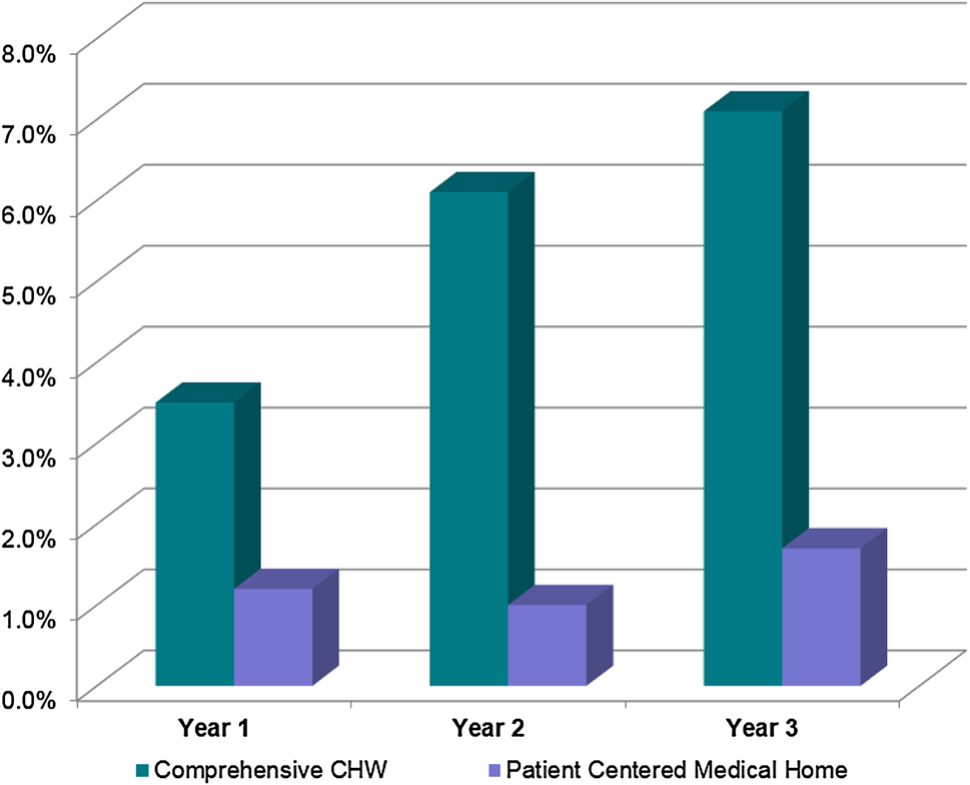
Anticipated savings from the Comprehensive CHW and Patient Centered Medical Model in primary care settings

The expected cost impact of the I-PACS CHW model suggests that hospital costs decrease approximately 70% for the high-risk patients, 40% for moderate risk individuals and 0% for healthy patients. Using the expected value assumptions above, we expected a decrease in emergency services of 61% for high-risk, 25% for moderate-risk, and 10% for low-risk patients. Salary cost for professional services is fixed at the primary care system level. So, anticipating increased utilization of services, the cost is projected to increase 20% for primary care and 10% for specialty care. Laboratory services are expected to increase with increased monitoring of clinical measures. The 5% increase in covered costs includes the increase due to CHW salary costs. In sum, the Figure projects the anticipated annual savings by the third year at 1.4% for the PCMH and 7.0% for the I-PaCS CHW model (Fig. 1).

## Discussion

Most studies find the bulk of PCMH cost savings are for high-risk, high resource-utilizing patients, showing fewer effects of the PCMH model on patients with moderate risk and utilizing and those with little risk and utilization [6]. Early studies demonstrated the cost effectiveness of CHWs working with high risk/high cost Medicaid Managed Care patients, reducing emergency department use and increasing primary care use [7, 8]. However, CHWs also address adverse SDH in moderate-risk, moderate resource-utilizing patients, yielding added cost saving benefits to the health system. By looking at cost savings at the practice and not only at the patient level, a far greater difference in cost savings is realized between the PCMH and I-PaCS CHW models. Thus, our estimates indicate that the PCMH and CHW models can be complementary, the latter helping the former realize a far greater cost savings.

## References

[1] McGinnis JM, Foege WH (1993). Actual causes of death in the United States. JAMA: The Journal of the American Medical Association.

[2] DeVoe JE, Bazemore AW, Cottrell EK, Likumahuwa-Ackman S, Grandmont J, Spach N, Gold R (2016). Perspectives in primary care: A conceptual framework and path for integrating social determinants of health into primary care practice. Annals of Family Medicine.

[3] Kaufman A (2016). Theory vs practice: should primary care practice take on social determinants of health now? yes. Annals of Family Medicine.

[4] Rosenthal MB, Alidina S, Friedberg MW, Singer SJ, Eastman D, Li Z, Schneider EC (2016). A difference-in-difference analysis of changes in quality, utilization and cost following the Colorado multi-payer patient-centered medical home pilot. Journal of General Internal Medicine.

[5] Maeng DD, Graham J, Graf TR, Liberman JN, Dermes NB, Tomcavage J, Davis DE, Bloom FJ, Steele GD (2012). Reducing long-term cost by transforming primary care: evidence from Geisinger’s medical home model. The American Journal of Managed Care.

[6] Altschuler J, Margolius D, Bodenheimer T, Grumbach K (2012). Estimating a reasonable patient panel size for primary care physicians with team-based task delegation. Annals of Family Medicine.

[7] Johnson D, Saavedra P, Sun E, Stageman A, Grovet D, Alfero C, Maynes C, Skipper B, Powell W, Kaufman A (2012). Community health workers and Medicaid managed care in New Mexico. Journal of Community Health.

[8] Mirambeau AM, Wang G, Ruggles L, Dunet DO (2013). A cost analysis of a community health worker program in rural Vermont. Journal of Community Health.

[9] Office of the Actuary at the Centers for Medicare and Medicaid Services. https://www.cms.gov/About-CMS/Agency-Information/CMSLeadership/Office_OACT.html. Accessed January 28, 2017.

